# Dietary Magnesium Intake and Leukocyte Telomere Attrition in Adults: The Regulatory Role of Serum Tumor Necrosis Factor *α*

**DOI:** 10.1155/2020/7610436

**Published:** 2020-05-22

**Authors:** Jie Yu, Haibin Liu, Shuli He, Pingping Li, Chunxiao Ma, Minglei Ma, Yiwen Liu, Lu Lv, Fan Ping, Huabing Zhang, Wei Li, Qi Sun, Lingling Xu, Yuxiu Li

**Affiliations:** ^1^Department of Endocrinology, Key Laboratory of Endocrinology, Ministry of Health, Peking Union Medical College Hospital, Beijing 100730, China; ^2^Department of Basic Physiology, The Health School Affiliated to Capital Medical University, China; ^3^Department of Nutrition, Peking Union Medical College Hospital, Beijing 100730, China; ^4^State Key Laboratory of Bioactive Substance and Function of Natural Medicines, Institute of Materia Medica, Chinese Academy of Medical Sciences & Peking Union Medical College, Beijing 100050, China; ^5^State Key Laboratory of Bioactive Substances and Functions of Natural Medicines, Beijing 100050, China

## Abstract

**Objectives:**

In this study, we assessed the effects of dietary magnesium on leukocyte telomere length (LTL).

**Designs:**

The current cross-sectional analysis was based on data collected within a type 2 diabetes project. *Settings*. Dietary magnesium intake is associated with peripheral blood leukocyte telomere length (LTL). However, few epidemiological studies have evaluated the effects of magnesium on LTL in the clinical setting. *Participants*. This cross-sectional analysis included 467 participants (34.8% men). *Measurements*. Serum blood lipid profile, HbA1c, oxidative stress, and proinflammatory mediator levels were measured. Detailed dietary data were obtained using a 24 h food recall. LTL was assessed using a real-time PCR assay. Regression models and simple regulatory models were used for data analysis.

**Results:**

There was an inverse relationship between dietary magnesium and LTL (*P* < 0.001), with a between-extreme-quarter difference of -0.55. Conversely, there was a positive relationship between dietary magnesium and serum tumor necrosis factor (TNF) *α*, with an interquarter difference of 3.79 pmol/mL (*P* for trend = 0.006). Multivariate regression analysis revealed that the odds ratios (ORs) for shorter LTL and higher serum TNF*α* increased with magnesium intake, and the ORs of the differences between extreme quartiles were 2.60 (95% confidence interval (CI): 1.31–5.36; *P* = 0.003) and 1.98 (95% CI: 1.09–3.59; *P* = 0.008). There was a direct negative effect of dietary magnesium intake on LTL (*B* = −0.002; *P* = 0.001), which appeared to be indirectly influenced by TNF*α* (-0.002 to -0.0005).

**Conclusions:**

Dietary magnesium intake may be a critical component of the cellular aging process, and its effect could be partly mediated by TNF*α*.

## 1. Introduction

Telomeres are nucleoprotein complexes composed of guanine-rich TTAGGG repeats located at the ends of chromosomes in eukaryotic cells and are essential for maintaining cellular chromosome integrity [1]. Telomere attrition is one of the hallmarks of cellular aging [2]. Moreover, peripheral blood leukocyte telomere length (LTL) has recently been shown to be a useful biomarker for cellular aging.

In our previous study, LTL was found to be influenced by consumption of different foods, such as nuts, fish, seaweed, and sweetened carbonated beverages [3]. Furthermore, recent studies have suggested that dietary micronutrients may be associated with TL [4]. Magnesium is a crucial element that is required for the regulation of cellular and metabolic reactions. Alternations in magnesium content or its distribution within the body are associated with several age-related diseases, such as diabetes [5, 6]. Moreover, the effect of magnesium on cellular aging may be related to its interactions with telomere homeostasis [7].

The most well-studied indices of cellular stress involved in accelerated telomere attrition rates are inflammation and reactive oxygen species. Previous studies have indicated that activation of tumor necrosis factor *α* (TNF*α*) signalling is linked to shorter LTL [8]. Levels of magnesium in the body have been found to correlate with the degree of inflammation. Existing literature has demonstrated an inverse correlation between high levels of magnesium intake and circulating C-reactive protein [9]. Based on these studies, higher magnesium consumption may promote lower rates of LTL attrition. However, data to support this potential correlation are conflicting. The National Health and Nutrition Examination Survey (NHANES) carried out in the United States of America demonstrated that increased dietary intake of magnesium increased LTL across all quartiles of intake levels [10]. Conversely, a 2014 paper published by O'Callaghan et al. reported negative correlations between plasma magnesium levels and telomere length, particularly in older women [11]. Experimental *in vivo* studies also yielded contrasting conclusions. Researchers found that elderly rats that had a 2-year history of consuming a diet moderately deficient in magnesium (0.15 g magnesium/kg diet) exhibited shorter telomere lengths in their livers compared with the other groups. These findings were attributed to lower rates of oxidative stress and apoptosis [12]. However, Kurstjens et al. showed that a lower dietary magnesium intake ameliorated high-fat diet-induced obesity in mice [13]. Thus, more studies are necessary to clarify the relationship between dietary magnesium intake and LTL.

Accordingly, in this study, we analysed the relationships among dietary magnesium intake, LTL, and the serum inflammation marker TNF*α*.

## 2. Materials and Methods

### 2.1. Subjects

This cross-sectional analysis used data previously collected from a type 2 diabetes mellitus cohort consisting of 599 participants enrolled between March 2014 and January 2015 in a suburb of Beijing [3]. Participants provided written informed consent for participation in the study. Information regarding basic demographic characteristics, medical history, and dietary intake were collected. All participants underwent a physical examination. An oral glucose tolerance test (OGTT) was carried out after an overnight fast (>10 h). Blood samples were collected at 0, 30, 60, and 120 min. The 1999 World Health Organization criteria were used to define the glucose tolerance status as follows: normal glucose tolerance (NGT) was defined as having a fasting plasma glucose (FPG) level of less than 6.1 mmol/L and a 2 h postprandial glucose (2 h PG) level of less than 7.8 mmol/L; prediabetes was defined as impaired glucose tolerance, with an FPG level of less than 6.1 mmol/L and a 2 h PG level between 7.8 and 11.1 mmol/L, and/or impaired fasting glucose, with an FPG level between 6.1 mmol/L and 7.0 mmol/L and a 2 h PG of less than 7.8 mmol/L; diabetes was defined as having an FPG level of greater than or equal to 7.0 mmol/L or a 2 h PG of greater than or equal to 11.1 mmol/L.

Participants who have impaired renal function, who are taking drugs or supplements that could affect magnesium intake and blood levels, and who did not have LTL or TNF*α* data were not included in the study. Thus, 467 individuals were finally included in the analysis, including 187 individuals with NGT, 146 individuals with prediabetes, and 134 individuals with diabetes mellitus. The study protocol was approved by the Ethics Committee of Peking Union Medical College Hospital.

### 2.2. Anthropometric Measurements

Blood pressure was determined using a standardised mercury sphygmomanometer with the participant seated. The body mass index was derived by dividing the body weight in kilograms by the square of the height in meters. Waist circumference was measured to the nearest 0.1 cm halfway between the iliac crest and the costal margin. Central obesity was defined as a waist circumference of greater than or equal to 90 cm in men and 85 cm in women.

### 2.3. Biochemical Tests

Blood samples were analysed immediately or kept at -80°C until further analysis. Haemoglobin (HbA1c) analysis was performed by high-performance liquid chromatography (intra-assay coefficient of variation (CV) < 3%, interassay CV < 10%). A glucose oxidase assay was used to measure plasma glucose. An automated analyser was used to determine low-density lipoprotein cholesterol, high-density lipoprotein cholesterol (HDL-C), triglyceride (TG), and total cholesterol. TNF*α* levels were analysed using a kit (Cloud-Clone Corp., Houston, TX, USA), according to the manufacturer's instructions.

### 2.4. Measurement of LTL

Peripheral blood LTL analysis was performed as previously described [3]. In brief, LTL was determined as the relative ratio of telomere repeat copy number to single copy number (T/S) using the novel monochrome multiplex quantitative PCR protocol described by Cawthon [14]. The within-plate and between-plate CVs were 8.2-14.3% and 7%, respectively.

### 2.5. Dietary Assessment

Dietary information was collected using 24 h food recalls. The dietary data were reviewed by dietitians and entered into nutrition calculation software (developed by researchers based on a Microsoft Office Access 2007 database). Data regarding food ingredients were obtained using the China Food Composition Table (2004) database as a guide.

### 2.6. Statistical Analysis

Normally distributed quantitative variables were reported as means and standard deviations (SDs) or standard errors (SEs). Parameters that were not normally distributed were first transformed, with categorical data presented as percentages or ratios. Chi-squared tests (for categorical variables) or a general linear model (GLM, for continuous variables) were used to compare participant characteristics between groups that had long or shorter LTLs (the lowest quartile of LTL was interpreted as having the shorter value). The LTL data were adjusted for age and sex. Dietary magnesium intake per day was adjusted for total energy intake, and magnesium intake per 1000 kcal energy was also calculated.

According to the quartiles of magnesium intake per 1000 kcal energy, the associations of magnesium intake with TNF*α* and LTL were analysed using a GLM. Model 1 was adjusted for sex and age, whereas model 2 was adjusted for central obesity, hypertension, HbA1c, LnTG, and HDL-C.

Magnesium intake was presented in terms of quartiles. The reference group was designated as those in the lowest quartile. Both LTL and TNF*α* were modelled as quartiles, and the highest quartile of TNF was interpreted as having the higher value, whereas the lowest quartile of LTL was interpreted as having the lower value. The association between the dichotomised levels of LTL and TNF*α* across the quartiles of magnesium intake was analysed via logistic regression. Linear trend tests across quartiles were evaluated by interpreting model quartiles as a continuous variable. The risks of increased TNF*α* and decreased LTL related to a 1 SD change in magnesium intake were also determined.

To investigate whether TNF*α* modulated the relationship between magnesium and LTL, macro PROCESS version 3.3 was used to generate simple regulatory models using ordinary least squares. These models were adjusted for sex, age, central obesity, hypertension, HbA1c, LnTG, and HDL-C. All variables were modelled as continuous variables. Regulatory hypotheses were tested via a bias-corrected bootstrap method with 5000 samples to calculate confidence intervals (95%). Significance was achieved when an indirect effect was observed, and zero was not included in the confidence intervals.

SPSS, version 25.0 (IBM Corp., Chicago, IL, USA) was used to perform all statistical analyses. All *P* values were two-tailed, and results with *P* values of less than 0.05 were considered significant.

## 3. Results

### 3.1. General Characteristics of the Participants

Table 1 depicts the clinical characteristics of all participants in this study. In general, magnesium intake by the study population was low, with only about 19.7% of participants having a dietary magnesium intake above 310 mg/day. Participants with the shortest LTL had higher serum TNF*α* levels and higher dietary intake of magnesium (Table 1). Moreover, participants with the shortest LTL had higher caloric intake, although this result was not significant, and had a higher carbohydrate and protein intake but less fat intake compared with other participants (Table 1). There were no significant differences among other metabolic parameters between the two groups (Table 1).

### 3.2. Relationships of Dietary Magnesium with LTL and TNF*α*

LTL and dietary magnesium were found to have a significant inverse relationship (*P* for trend < 0.001) after adjusting for variables, with a between-extreme-quartile difference of -0.55 (Table 2). In contrast, serum TNF*α* was positively correlated with dietary magnesium intake, with participants in the highest quartile of magnesium consumption (Q4) also having the highest TNF*α* levels (Q4). There was an interquartile difference of 3.79 pmol/mL (*P* for trend = 0.006), equivalent to 17.6% of Q1 (Table 2).

In order to further identify the associations of dietary magnesium with LTL and TNF*α*, multivariate logistic regression analyses were used, as shown in Table 3. Both LTL and TNF*α* were modelled as quartiles, and the highest quartile of TNF was interpreted as having the higher value, whereas the lowest quartile of LTL was interpreted as having the lower value. After adjusting for age, sex, central obesity, hypertension, HbA1c, LnTG, and HDL-C, the odds ratios (ORs) of shorter LTL were higher as dietary magnesium intake increased, and the OR for the comparison of the two extreme quartiles was 2.60 (95% CI: 1.31–5.36; *P* for trend = 0.003). The OR (95% CI) for shorter LTL per 1 SD increment in magnesium intake was 1.49 (95% CI: 1.16–1.92). For TNF*α*, the ORs of higher serum TNF*α* also increased as magnesium intake increased, and the OR for the comparison between the extreme quarters was 1.98 (95% CI: 1.09–3.59; *P* for trend = 0.008). The OR (95% CI) for higher serum TNF*α* per 1 SD increment in magnesium intake was 1.25 (95% CI: 1.02–1.52).

### 3.3. TNF*α* Significantly Regulated the Association between Magnesium and LTL

**Figure 1:**
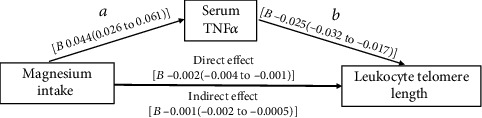
TNF*α* as mediator of the effects of magnesium intake on LTL. All variables were modeled as continuous variables. The model was adjusted according to age, sex, central obesity, hypertension, HbA1c, LnTG, and HDL-C.

Figure 1 depicts the regulatory model used to determine the involvement of TNF*α* in facilitating the deleterious effects of magnesium intake on LTL. Regression a (*B* = 0.044; *P* < 0.001) indicated that magnesium was positively associated with higher TNF*α*, and regression b (*B* = −0.025; *P* < 0.001) demonstrated that there was a significant association between shorter LTL and higher TNF*α*. Additionally, there appeared to be a direct link between magnesium intake and LTL (*B* = −0.002; *P* = 0.001). Our working hypothesis was confirmed as the CIs for indirect effect did not involve the number zero (-0.002 to -0.0005). In summary, TNF*α* appeared to play a role in mediating the effects of magnesium intake on LTL attrition.

### 3.4. Contribution of Different Foods to Magnesium Intake

**Figure 2:**
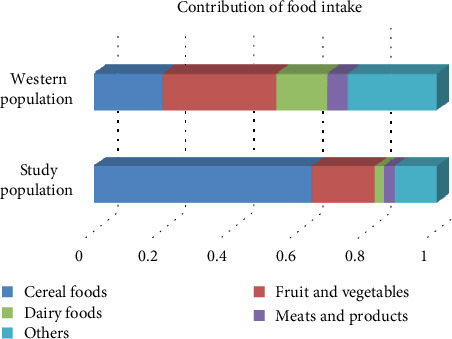
Contributions of different foods to magnesium intake in our study population and a western population. The data of the above bar were adopted and slightly modified from an article by Welch et al. [15]. The data of below the bar were generated from our study population.

In our population, 87.8% of magnesium intake was derived from fruit and vegetables (18.3%), cereal foods (63.7%), dairy foods (2.7%), and meats and meat products (3.1%). These values were distinctly different from those in the western population (Figure 2) [15].

## 4. Discussion

In this study, we demonstrated the inverse relationship between dietary magnesium and LTL and showed that increased levels of the serum inflammation marker TNF*α* could be a risk factor for LTL attrition. Pathway analysis indicated that serum TNF*α* levels facilitated the association between higher dietary magnesium intake and shorter LTL.

We focused on dietary magnesium and its relationship with telomere shortening because diet represents an easily modifiable intervention target. Our findings demonstrated a negative correlation between LTL and magnesium intake across different types of analyses adjusted according to several potential confounding factors. Although some studies have explored the associations between mineral micronutrients (e.g., iron, copper, and zinc) and LTL, only two cross-sectional studies have reported the association between magnesium and LTL. One study by Mazidi et al. reported a positive relationship between dietary intake of magnesium and LTL in the NHANES study [10]. The other one showed that older women had relatively higher serum magnesium levels and shorter LTLs [11], which somehow supported our results, as serum magnesium was positively associated with dietary magnesium intake.

Furthermore, simple regulatory models showed that dietary magnesium intake was correlated with LTL attrition risk both directly and indirectly through modification of serum TNF*α* levels. Inflammation may have a role in modulating the effects of dietary magnesium intake on LTL shortening. However, our results were inconsistent with previous studies demonstrating a negative relationship between magnesium and inflammation. These prior studies postulated that magnesium may have protective effects on telomere attrition and aging [7, 16].

The differences between our results and previous studies may be attributed to several reasons. Firstly, the inherent population characteristics were different. As with LTL, alterations in magnesium homeostasis are known to be associated with several age-related diseases, such as metabolic syndrome and type 2 diabetes. Most studies with populations from Western countries have shown that serum magnesium levels were lower in patients with metabolic syndrome than in controls [17]. Additionally, dietary magnesium intake has been shown to have an inverse correlation with the risk of metabolic syndrome [18]. However, several studies in Asian populations have demonstrated that serum magnesium levels are increased in patients with metabolic syndrome, obesity, hypertension, or hyperlipidaemia [19–22]. One matched case-control study of patients with metabolic syndrome showed that the ORs for metabolic syndrome increased to 1.812 and 1.923 for the median tertile and top tertile of serum magnesium, respectively, compared with those in the bottom tertile [21]. Secondly, the different results between Asian and Western populations may be related to differences in diet. In contrast to Western populations, participants in our study consumed high levels of carbohydrates but low levels of fats, with cereals accounting for the majority of magnesium intake [15]. Magnesium may exist in different forms depending on the food source, thus resulting in different absorption efficiencies and physiological functions. Finally, differences between our results and previous studies may be related to variations in research design and study heterogeneity. More rigorously designed cohort studies are needed to confirm our conclusions.

## 5. Strengths and Limitations

One of the strengths of this study was that the project cohort was highly representative of the population with different glucose status. However, there were still several limitations to this study. First, this was a cross-sectional study, which eliminated our ability to determine causality. Such studies are only able to identify associations, thus laying the groundwork for additional studies. Furthermore, although we included participants having several different plasma glucose levels, the sample size was relatively small. Another limitation was the inability to rule out recall biases despite trained investigators and an extensive dietary composition investigation questionnaire based on the Chinese food ingredient scale.

## 6. Conclusions

In conclusion, our results indicated that there was an inverse association between dietary magnesium intake and peripheral blood LTL. By using a simple regulatory model, we found a significant indirect effect of serum TNF*α* on the link between LTL and dietary magnesium intake. Dietary magnesium intake may have negative effects on LTL shortening through promotion of inflammation. Differences in ethnicities, diets, and study heterogeneity necessitate further studies to confirm our findings. Thus, future large-scale prospective cohort studies measuring both dietary magnesium intake and serum magnesium in individuals of different ethnicities are needed.

## Figures and Tables

**Table 1:** Characteristics and dietary intake of participants by quartiles according to LTL.

Parameters	Overall	Shortest LTL (*n* = 117)	Longer LTL (*n* = 350)	*P* value
^a^LTL	28.33 ± 0.03	27.61 ± 0.05	29.04 ± 0.03	<0.001^∗^
Male (*n*, %)	164 (34.8%)	39 (33.1%)	125 (35.4%)	0.641
Age (years)	52.8 ± 11.4	53.7 ± 10.8	52.5 ± 11.6	0.308
BMI (kg/m^2^)	26.14 ± 0.28	25.80 ± 0.48	26.48 ± 0.28	0.218
Central obesity (*n*, %)	244 (52.9%)	61 (52.6%)	183 (53.0%)	0.932
Hypertension (*n*, %)	233 (50.2%)	63 (53.8%)	170 (49.0%)	0.364
HbA1c (%)	272 (59.6%)	66 (58.4%)	206 (60.1%)	0.756
LnTG (mmol/L)	0.39 ± 0.63	0.38 ± 0.54	0.41 ± 0.65	0.673
LDL-C (mmol/L)	2.87 ± 0.73	2.86 ± 0.76	2.87 ± 0.72	0.941
HDL-C (mmol/L)	1.31 ± 0.41	1.34 ± 0.56	1.30 ± 0.34	0.37
TNF*α* (pmol/mL)	23.86 ± 10.44	28.34 ± 9.23	22.34 ± 10.40	<0.001^∗^
Energy (kcal/day)	1579.39 ± 698.49	1651.6 ± 730.03	1555.77 ± 687.39	0.225
Protein (% energy)	11.2% ± 3.3%	11.8% ± 3.2%	11.0% ± 3.3%	0.038^∗^
Fat (% energy)	25.0% ± 16.1%	21.9% ± 13.6%	26.0% ± 16.8%	0.027^∗^
Carbohydrate (% energy)	65.8% ± 16.6%	69.2% ± 17.1%	64.6% ± 16.3%	0.014^∗^
^b^Magnesium (mg/day)	247.13 ± 10.29	278.18 ± 17.86	216.08 ± 10.21	0.003^∗^
^c^Magnesium (mg/day)	242.67 ± 7.33	264.93 ± 12.73	220.42 ± 7.28	0.003^∗^
Magnesium (mg/1000 kcal)	149.75 ± 3.19	160.66 ± 5.54	138.85 ± 3.17	0.001^∗^

^∗^Statistical significance was inferred when the *P* value was less than 0.05. ^a^LTL adjusted according to age and sex and expressed as mean ± SE. ^b^Unadjusted magnesium intake, expressed as mean ± SD. ^c^Magnesium intake adjusted according to energy intake, expressed as mean ± SE; other continuous variables were expressed as means ± SDs.

**Table 2:** LTL and TNF*α* by quartiles according to magnesium intake per 1000 kcal energy.

	Model	Q1 (*n* = 117)	Q2 (*n* = 117)	Q3 (n = 117)	Q4 (*n* = 116)	*P* for trend	Q4–Q1	% of Q1
Mg		89.20 ± 2.77	120.43 ± 2.77	153.05 ± 2.77	237.22 ± 2.77	—	—	—
LTL	1	28.89 ± 0.08	28.79 ± 0.08	28.69 ± 0.80	28.36 ± 0.09	<0.001^∗^	-0.53	-1.8
	2	28.90 ± 0.08	28.80 ± 0.08	28.71 ± 0.08	28.35 ± 0.09	<0.001^∗^	-0.55	-1.9
TNF*α*	1	21.51 ± 0.91	21.45 ± 0.93	23.72 ± 0.93	25.53 ± 0.92	0.004^∗^	4.02	18.9
	2	21.54 ± 0.93	21.07 ± 0.95	23.59 ± 0.92	25.33 ± 0.95	0.006^∗^	3.79	17.6

^∗^Statistical significance was inferred when the *P* value was less than 0.05; results are depicted as means ± SEs; ANCOVA was used to derive the *P* for trend values. Q4–Q1 is the value in Q4 minus the value in Q1. % of Q1 represents the difference between Q4 and Q1 with respect to the Q1 value. Model 1 was adjusted for sex and age, whereas Model 2 was adjusted for hypertension, central obesity, sex, age, HbA1c, LnTG, and HDL-C.

**Table 3:** Odds ratios and 95% CIs for lowest LTL and highest TNF*α* across Mg intake.

	Q1	Q2	Q3	Q4	*P* for trend	1SD increment
	Ref.	OR (95% CI)	OR (95% CI)	OR (95% CI)		OR (95% CI)
LTL	1	1.17 (0.57–2.38)	1.76 (0.90–3.43)	2.60 (1.31–5.16)	0.003^∗^	1.49 (1.16–1.92)
TNF*α*	1	0.83 (0.43–1.58)	1.29 (0.71–2.37)	1.98 (1.09–3.59)	0.008^∗^	1.25 (1.02–1.52)

^∗^Statistical significance was inferred when the *P* value was less than 0.05; models were adjusted according to age, sex, central obesity, hypertension, HbA1c, LnTG, and HDL-C.

## Data Availability

Data generated or analyzed during this study could be available by asking the corresponding authors.
