# Corrigendum to “Dietary Magnesium Intake and Leukocyte Telomere Attrition in Adults: The Regulatory Role of Serum Tumor Necrosis Factor *α*”

**DOI:** 10.1155/2020/2594730

**Published:** 2020-07-18

**Authors:** Jie Yu, Haibin Liu, Shuli He, Pingping Li, Chunxiao Ma, Minglei Ma, Yiwen Liu, Lu Lv, Fan Ping, Huabing Zhang, Wei Li, Qi Sun, Lingling Xu, Yuxiu Li

**Affiliations:** ^1^Department of Endocrinology, NHC Key Laboratory of Endocrinology, Peking Union Medical College Hospital, Chinese Academy of Medical Sciences and Peking Union Medical College, Beijing 100730, China; ^2^Department of Basic Physiology, The Health School Affiliated to Capital Medical University, China; ^3^Department of Nutrition, Peking Union Medical College Hospital, Beijing 100730, China; ^4^State Key Laboratory of Bioactive Substance and Function of Natural Medicines, Institute of Materia Medica, Chinese Academy of Medical Sciences & Peking Union Medical College, Beijing 100050, China

In the article titled “Dietary Magnesium Intake and Leukocyte Telomere Attrition in Adults: The Regulatory Role of Serum Tumor Necrosis Factor *α*” [1], authors “Jie Yu, Minglei Ma, Yiwen Liu, Lu Lv, Fan Ping, Huabing Zhang, Wei Li, Qi Sun, Lingling Xu, and Yuxiu Li” are affiliated to “^1^Department of Endocrinology, Key Laboratory of Endocrinology, Ministry of Health, Peking Union Medical College Hospital, Beijing 100730, China” which is incorrect. The correct affiliation for these authors is as follows:

Department of Endocrinology, NHC Key Laboratory of Endocrinology, Peking Union Medical College Hospital, Chinese Academy of Medical Sciences and Peking Union Medical College, Beijing 100730, China.

The corrected list of affiliations is shown in the author information above.
